# Gaseous Colic

**Published:** 1856-09

**Authors:** W. T. Grant

**Affiliations:** Wrightsboro’, Columbia County, Georgia


*Gaseous Colic.* By W. T. GRANT, M. D., of Wrightsboro', Columbia county, Georgia.

I was called recently, in haste, to see a negro man 40 years of age, laboring under, as I was told, "cramp colic." On my arrival, I perceived at once, that the colic was gaseous. The history of the case, prior to my seeing it, was somewhat as follows: He had eaten a large quantity—perhaps a quart—of peas, which were half cooked, for breakfast, on the morning of the day of his sickness, and went immediately out into the field to work. About 10 o'clock, he complained to his overseer, that he was very sick, who told him to go home; which he did at once, the overseer going with him. The overseer tells me that when they reached the house, the negro was beginning to swell, and he gave him some camphor, which caused him to vomit, after which, he sank down upon the ground, losing, apparently, all his strength. The overseer then hurried off after a physician, and I reached the farm by eleven o'clock, or a little after. I found the negro enormously distended in the upper part of his body, reaching as low down as the umbilicus. The interspaces between the ribs were protruded, and he was as tight as a drum. He had no radial pulse, nor any other that I could perceive; respiration very feeble, and very slow; extremities cold. The heart and lungs were almost entirely impeded in the performance of their functions—so much, indeed, that I think he would have died in a short time if relief had not been afforded.

The indication, and the only indication, was to get rid of the gas. I noticed that, whenever he vomited, it brought up some of the gas. I administered an emetic, which acted promptly, and brought up a considerable quantity, but all that was brought up by this means, was so little, in comparison with the quantity that was in him, that it produced no relief. I thought, then, that by distributing the gas through his bowels, it would relieve his heart and lungs until I could adopt such means as was calculated to rid him of the gas entirely. I accordingly administered a cathartic; but that was vomited up immediately. It then occurred to me to use the *pulverized charcoal*, (carbo ligni,) and I had some of the coals from the fireplace pulverized, and given him, which soon indicated a favorable change, and in two hours, he was so much relieved that I considered him out of danger. Soon after its administration, the pulse began to rise, and in a short time, was comparatively a good pulse. After directing that one or two doses of the charcoal should be given him during the evening, to insure an entire absorption of the gas, and at night, a good dose of castor oil, I left him entirely free from all danger. The next day he was well, with the exception of the necessary soreness of the abdomen and chest, and some debility. I presume that the *modus operandi* of the charcoal was absorption of the gas.

I should have mentioned, that when the overseer gave him the camphor and caused him to vomit, he brought up a quantity of peas entirely undigested, and swollen as large as the end of a man's thumb.

